# The Effect of Chitosan/Alginate/Graphene Oxide Nanocomposites on Proliferation of Mouse Spermatogonial Stem Cells

**DOI:** 10.3390/jfb14120556

**Published:** 2023-11-22

**Authors:** Alaa Moeinzadeh, Behnaz Ashtari, Heriberto Garcia, Morteza Koruji, Carlo Alberto Velazquez, Zohreh Bagher, Mahmood Barati, Ronak Shabani, Seyed Mohammad Davachi

**Affiliations:** 1 Department of Anatomy, School of Medicine, Iran University of Medical Sciences, Tehran, Iran; 2 Department of Tissue Engineering and Regenerative Medicine, Faculty of Advanced Technologies in Medicine, Iran University of Medical Sciences, Tehran, Iran; 3 Radiation Biology Research Center, Iran University of Medical Sciences, Tehran, Iran; 4 Department of Medical Nanotechnology, Faculty of Advanced Technologies in Medicine, Iran University of Medical Sciences, Tehran, Iran; 5 Department of Biology and Chemistry, Texas A&M International University, Laredo, TX 78041, USA; 6 Stem Cell and Regenerative Medicine Research Center, Iran University of Medical Sciences, Tehran, Iran; 7 ENT and Head & Neck Research Center and Department, The Five Senses Institute, Hazrat Rasoul Akram Hospital, Iran University of Medical Sciences, Tehran, Iran; 8 Department of Medical Biotechnology, Faculty of Allied Medicine, Iran University of Medical Sciences, Tehran, Iran; 9 Reproductive Sciences and Technology Research Center, Iran University of Medical Sciences, Tehran, Iran

**Keywords:** spermatogonial stem cells, nanocomposite scaffolds, graphene oxide, alginate, chitosan

## Abstract

Male survivors of childhood cancer have been known to be afflicted with azoospermia. To combat this, the isolation and purification of spermatogonial stem cells (SSCs) are crucial. Implementing scaffolds that emulate the extracellular matrix environment is vital for promoting the regeneration and proliferation of SSCs. This research aimed to evaluate the efficiency of nanocomposite scaffolds based on alginate, chitosan, and graphene oxide (GO) in facilitating SSCs proliferation. To analyze the cytotoxicity of the scaffolds, an MTT assay was conducted at 1, 3, and 7 days, and the sample containing 30 µg/mL of GO (ALGCS/GO30) exhibited the most favorable results, indicating its optimal performance. The identity of the cells was confirmed using flow cytometry with *C-Kit* and *GFRα1* markers. The scaffolds were subjected to various analyses to characterize their properties. FTIR was employed to assess the chemical structure, XRD to examine crystallinity, and SEM to visualize the morphology of the scaffolds. To evaluate the proliferation of SSCs, qRT-PCR was used. The study’s results demonstrated that the ALGCS/GO30 nanocomposite scaffold exhibited biocompatibility and facilitated the attachment and proliferation of SSCs. Notably, the scaffold displayed a significant increase in proliferation markers compared to the control group, indicating its ability to support SSC growth. The expression level of the *PLZF* protein was assessed using the Immunocytochemistry method. The observations confirmed the qRT-PCR results, which indicated that the nanocomposite scaffolds had higher levels of *PLZF* protein expression than scaffolds without GO. The biocompatible ALGCS/GO30 is a promising alternative for promoting SSC proliferation in in vitro applications.

## 1. Introduction

Male fertility depends on the foundation of spermatogonial stem cells (SSCs), which are essential for reproduction; maintaining an equilibrium between self-renewal and differentiation is critical for sustaining virility [1,2]. SSCs are found in seminiferous tubules near the basement membrane [3,4]. One stem cell could produce 1024 spermatocytes, and it has been estimated that about 75–90% of spermatocytes undergo apoptotic cell death during meiotic cell division. Thus, the remaining cells are only about 0.03%, which is very low compared to testicular germ cells [5,6]. The spermatogonial proliferation phase is characterized by the significant increase in germ cells count, which serves as a primary mechanism underlying the productivity of spermatogenesis [7,8]. Azoospermia, a complication in male survivors of childhood cancer, highlights the importance of the pre-treatment isolation and purification of SSCs. Accordingly, culturing these cells is very important, especially for children who had cancer before puberty and were affected by chemotherapy, radiotherapy, and other anti-cancer treatments [9,10,11]. The current understanding suggests that the conventional 2D system does not provide the optimal conditions for supporting the complex structure of seminiferous tubules, which are essential for spermatogenesis. In the field of tissue engineering (TE), three-dimensional (3D) scaffolds play a pivotal role by offering physical and structural support to cells while creating a microenvironment that closely resembles the native extracellular matrix [12,13,14]. The utilization of 3D systems has opened up a promising avenue for treating male infertility by facilitating the proliferation and differentiation of SSCs. [15]. Various materials have been used for fabricating the scaffolds, with synthetic polymers, such as polyglycolic acid (PGA), polylactic acid (PLA), poly glycerol sebacate (PGS), polycaprolactone (PCL), poly-L-lactic acid (PLLA), and polypropylene fumarate (PPF), being at the forefront [16,17,18]. Despite their wide use, synthetic polymers have certain drawbacks in tissue engineering applications. These drawbacks include limited cellular attachment caused by hydrophobic nature and the potential generation of toxic byproducts during in vivo biodegradation [19,20].

Recently, there has been increasing interest in utilizing natural polymers such as alginate and chitosan as scaffolds for TE applications [21]. Alginate is an anionic polymer isolated from seaweed. It consists of a 1/4 linked β-D-mannuronic acid (M) and α-L-guluronic acid (G) structure [22]. Alginate possesses several advantageous properties for biomedical applications. Alginate is biocompatible, hydrophilic, biodegradable [23], cost-effective [24], and non-antigenic [25,26,27]. Chitosan is a biopolymer derived from the exoskeleton of crustaceans. It consists of (1-4)-2-acetamido-2-deoxy-β-D-glucan (N-acetyl D-glucosamine) and (1-4)-2-amino-2-deoxy-β-D-glucan (D-glucosamine) units [28]. It has high absorption capacity and is bioactive, biodegradable, biocompatible, non-toxic, non-antigenic, and anti-bacterial [29,30,31,32]. The physically woven structure of alginate (ALG) and chitosan (CS) offers a scaffold structure with enhanced mechanical properties, making it a promising candidate for applications in regenerative medicine and tissue engineering [33,34].

Graphene oxide (GO) is a single layer of sp^2^-bonded carbon atoms, hydrogen, and oxygen molecules produced by the oxidation of graphite crystals [35,36]. It has a unique structure, high thermal solubility, interfacial interactivity with a target matrix, hydrophilicity of the scaffold, and electrical conductivity [37,38,39]. Several studies have demonstrated that GO can modulate cellular behavior through its superior mechanical properties and substantial surface area within the polymer matrix [40,41,42]. However, only a few reports have been published regarding the success of GO in the enhancement of spermatogonial stem cell proliferation. Hashemi et al. assessed the cyto- and genotoxicity of GO on SSCs and observed that at high concentrations of GO (more than 100 µg/mL), cell toxicity increases. The suggested mechanism of cytotoxic effect was mitochondrial activity impairment, plasma membrane damage, the induction of oxidative stress, DNA damage, and apoptotic and/or necrotic cell death. However, the toxicity can be reduced by the surface modification of GO [43,44]. Carbon nanotubes (CNTs) have a similar chemical structure to GO and are a suitable substrate for storing spermatogonia stem cells in vitro [45]. It has been reported that using CNTs enhances the proliferation of SSCs [46]. In addition, it was found that the increase in surface roughness due to the presence of CNT plays a significant role in influencing the behavior of human bone marrow cells, including adhesion, proliferation, and differentiation [46]. Studies have reported that CNT can enhance the expression of *C-Kit* and SYCP3 proteins associated with spermatogenesis. These results suggest that CNT may positively impact the process of sperm cell development [47]. Due to their similarities in chemical structure, we believe that GO can act the same as CNT and enhance sperm cell development.

There is sufficient confirmation about the constructive impact of alginate and chitosan (ALGCS) on different types of cells, such as bone, cartilage, skin, cancer, and wound-healing cells, among others [48,49,50,51,52,53,54]. However, there are few studies on their usage with SSCs. Hemadi et al. used alginate hydrogels to improve the long-term culture of SSCs. They studied the gene expression and characterized the morphology and structure of the SSCs in the encapsulated alginate hydrogels. Their findings confirmed the formation of nucleolus colonies, while alginate preserved the morphology and density of the SSCs for more than 60 days [55]. Pirnia et al. reported that injection of freeze–thawed SSCs encapsulated in alginate hydrogel could successfully recover spermatogenesis since alginate can mimic the extracellular matrices (ECMs) for SSCs, protect the stemness of the SSCs during the cryopreservation process, and restart spermatogenesis after transplantation [56]. Jalayeri et al. used alginate hydrogels for SSCs culture and reported that alginate provides a non-toxic environment with adequate nutrition and oxygen support and does not interfere with cell viability and morphology [57]. Naeemi et al. reported that hyaluronic acid/chitosan-based scaffolds can support SSCs differentiation and proliferation [58]. The 3D growth of fragmented seminiferous tubules in a hollow chitosan hydrogel cylinder as a bioreactor was used to induce spermiogenesis [59]. The behavior and differentiation of SSCs on 3D scaffold culture systems made of poly L-lactic acid (PLLA) [60], polyamide nanofibers [61], and PCL/gelatin nanofibers [62] were also investigated. All the studies confirmed that improving the quality of the culture condition improved the number and diameter of SSC colonies.

The combination of chitosan, alginate, and GO has not been used in tissue engineering studies of reproductive systems so far. Therefore, in the current study, according to the unique characteristics of nanocomposite scaffolds based on alginate and chitosan, which can simulate similar conditions to the natural environment of spermatogonia in testicular tissue, by adding GO to the scaffold, it can be used as a suitable substrate for the proliferation and cloning of these cells in vitro. Creating an effective in vitro culture system for high-efficiency SSCs multiplication and differentiation is an important objective in fertility preservation methods and in vitro spermatogenesis studies.

## 2. Materials and Methods

Dulbecco’s modified Eagle’s medium/F12 (DMEM/F12) and Dulbecco’s phosphate-buffered saline (DPBS), penicillin, streptomycin, and gentamycin were purchased from Gibco Life Technologies (Carlsbad, CA, USA). GDNF family receptor alpha-1 (*GFRα1*) and receptor tyrosine kinase (*C-Kit*) markers as well as paraformaldehyde (PFA) were bought from Merck (Darmstadt, Germany). Graphene Oxide (GO, CAS No.: 1034343-98-0), sodium alginate (ALG, weight average molecular weight of 750 KDa), chitosan (medium molecular weight, deacetylation degree of 85–95%), sodium hydrosulfite (Na_2_S_2_O_4_), potassium permanganate (KMnO_4_), phosphate-buffered saline (PBS) with pH 7.4 for 1 L solution tablets, ethanol, isopropanol, fetal bovine serum (FBS), fluorophore-conjugated secondary antibody, fluorescein isothiocyanate (FITC), and 4′,6-diamidino-2-phenylindole (DAPI) staining kit were all purchased from Sigma-Aldrich (St. Louis, MO, USA). A QuantiNova Reverse Transcription (QNRT) kit was bought from Qiagen (Hilden, Germany). N-terminal rabbit monoclonal anti-promyelocytic leukemia zinc-finger (*PLZF*) primary antibody (ab189849) was purchased from Abcam (Cambridge, UK). YTA SYBR Green qPCR MasterMix 2X was acquired from Yekta Tajhiz Azma (Tehran, Iran), and RNX-Plus solution was obtained from Sinaclone (Tehran, Iran).

### 2.1. Synthesis of GO and Preparation of Scaffolds

#### 2.1.1. Synthesis of GO

There are several methods for synthesizing GO, including the Hummer synthesis method [47,63]. In the process described, 1 g of graphite is added to 20 mL of 98% purity sulfuric acid. The mixture is then placed in an ice bath and stirred. At low temperatures, typically achieved in the ice bath, graphite undergoes complete dissolution in the sulfuric acid with the assistance of a magnet. After approximately 30 min of the previous step, 3 g of potassium permanganate is added slowly to the solution. As the potassium permanganate is added, the color of the solution changes to a sludge green color. The solution is allowed to react and mix for another 30 min, then 50 mL of deionized (DI) water is added dropwise to the solution. After a further 10 min, 100 mL of DI water is added to the stirring solution. After 30 min of stirring, 35 mL of hydrogen peroxide is added dropwise to the solution. After 24 h, the GO solution is removed from the heater stirrer. To facilitate the samples’ drying process, they were centrifuged, freeze-dried, and stored in a refrigerator at a temperature of −20 °C until completely frozen.

#### 2.1.2. Fabrication of Alginate/Chitosan Scaffolds

4 wt% CS was added to 2 wt% acetic acid and mixed until a homogenous solution was produced. Then, 4 wt% ALG solution was prepared and added to the CS solution. The solution was then mixed for 5 min to obtain a homogenous alginate/chitosan (ALGCS) solution. After the mixture, the ALGCS solution was cast in a 24-well cell culture plate and freeze-dried. After preparing the ALGCS solution, the solution was cross-linked by immersing it in a 0.2 M CaCl_2_ solution under vacuum for 10 min. Next, the cross-linked scaffolds were washed multiple times with DI water to remove excess CaCl_2_ salt. After washing, the scaffold was immersed in 70% ethanol for 1 h. Subsequently, the scaffolds were transferred to a sterile DPBS solution and placed on an orbital shaker for 12 h. The purpose of this was to facilitate the removal of any excess ethanol [64,65].

#### 2.1.3. Preparation of ALGCS/GO Scaffolds

In an ice bath, GO solutions with various concentrations (5, 15, 30, 45, and 75 µg/mL) were treated using a probe sonicator for 15 min at 150W (SONOPULS HD2070, Bandelin, Berlin, Germany). To activate the carboxyl group of GO, EDC was introduced into the GO solutions in water at a weight ratio of 1000:5:4. After stirring the mixtures for 15 min, the ALGCS solution was added in the prepared GO solutions and placed on a shaker at 25 °C for 6 h. The solution was titrated using 0.1 M hydrochloric acid to achieve a pH of 4.7. Following the reaction, the ALGCS/GO scaffolds underwent a washing step using DI water to remove EDC and salt residues that may be present on the scaffolds. After the washing step, the scaffolds were subjected to lyophilization. In addition to the previous steps, some scaffolds underwent a reduction process. This process involved immersing the scaffolds in 2% Na_2_S_2_O_4_ solution for 3 min, then rinsing with ethanol and air-drying [66]. Prepared samples were abbreviated as shown in Table 1. Optical micrographs of the prepared scaffolds shown in Supplementary Material Figure S1 confirmed that by increasing the concentration of GO, the color of ALGCS scaffolds becomes darker.

### 2.2. Characterization of the ALGCS Scaffolds

#### 2.2.1. FTIR

Fourier-transform infrared (FTIR) spectroscopy using Avatar 330 (Thermo Co., Waltham, MA, USA) was performed to conduct a structural analysis of the purified components and their interactions within the scaffolds. The resolution of the test was 4 cm^−1^ (averaging 128 scans) under transmittance mode in the 400–4000 cm^−1^ range.

#### 2.2.2. Scanning Electron Microscopy

The scaffold morphology was examined using field emission scanning electron microscopy (FESEM, MIRA III, TESCAN, Brno, Czech). Prior to the test, SEM samples underwent coating with a 15 nm layer of Au using the Denton Desk V sputter coater (Moorestown, NJ, USA).

#### 2.2.3. X-ray Diffraction

The crystallinity, chemical composition, and phase identification of the specimens were examined using X-ray diffraction (XRD) via X’Pert MPD Philips (Almelo, The Netherlands) equipped with an operating source of Cu-Kα radiation (λ = 1.79 A°) at a current of 40 mA and a voltage of 40 kV. The diffraction angle (2θ) was scanned from 5° to 80° at room temperature at a scanning rate of 0.05°/s.

#### 2.2.4. Mechanical Properties

The mechanical strength of the samples was studied by measuring the compression strength using an Instron 5960 tensile tester (Instron Co., Norwood, MA, USA). The scaffolds with a diameter of ~15 mm and a height of ~30 mm were assessed with a 100 kN load cell and a crosshead rate of 5 mm/min. The experiment was performed in triplicates, and the average ± standard deviations were reported.

#### 2.2.5. Swelling Studies

The swelling behavior of the lyophilized scaffolds was observed by immersing the samples in PBS (pH 7.4) at 37 °C and at different time intervals (0.5, 1, 2, 4, 6, 24, 48, and 72 h). Following incubation in PBS, the samples were cautiously removed, and any excess water on their surfaces was dried using sheets of paper. The swelling ratios were calculated using Equation (1), where W_w_ is the weight of the swollen scaffold, and W_d_ is the initial dried weight [67].

(1) $$\mathrm{Swelling}\;(\%)=\frac{W_{w}{\;-\;W}_{d}}{W_{w}}\times 100$$

#### 2.2.6. Hydrolytic Degradation

The samples were placed in PBS, and their degradation was assessed at defined intervals (3, 7, and 14 days). Weight loss percentage was calculated using Equation (2), where m_0_ is the scaffolds’ initial weight, and mL is the scaffolds’ final mass [67].

(2) $$\mathrm{Weight}\;\mathrm{loss}\;(\%)=\;\frac{M_{0}{\;-\;M}_{L}}{M_{0}}\times 100$$

#### 2.2.7. Measurement of Porosity

Isopropanol was selected as a solvent to evaluate the porosity of the scaffolds due to its ability to penetrate the scaffold’s pores without causing significant shrinkage or swelling. The dry scaffolds were immersed in isopropanol for 24 h in the vacuum oven at 25 °C. After the immersion period, the samples were carefully removed from the isopropanol solution. Excess isopropanol on the surfaces of the samples was gently removed using filter paper to facilitate drying. Then, the samples were weighed to determine their weight after the isopropanol treatment [68]. Porosity was calculated from Equation (3), where W_d_ is dry weight, W_w_ is wet weight, and W_i_ is immersed weight [68].

(3) $$\mathrm{Porosity}\;(\%)=\;\frac{W_{w}{\;-\;W}_{d}}{W_{w}{\;-\;W}_{i}}\;\times \;100$$

### 2.3. Cell Culture and In Vitro Studies

#### 2.3.1. Isolation of Mouse Spermatogonial Stem Cells

Eighty neonatal (3–6 days old) NMRI mice provided by Iran University of Medical Sciences (Tehran, Iran) were used for this examination. The mice involved in the study were housed in cages constructed from plastic material. The housing was kept at a 22–25 °C temperature range and followed a 12 h light/dark cycle. Following the Animal Ethic Committee of the Iran University of Medical Science, the mice had unrestricted access to drinking water and standard laboratory pellets. (Code: IR.IUMS.FMD.REC.1399.188. Date: 14 June 2020). Initially, testes of the neonatal mice were collected bilaterally. A 2-stage enzymatic digestion protocol was carried out to obtain a single-cell suspension. After decapsulation, the testes were dissected into small pieces and suspended in Dulbecco’s Modified Eagle’s Medium/F12 supplemented with penicillin (100 IU/mL), gentamycin (40 µg/mL), and streptomycin (100 µg/mL). To prepare the first stage of the enzyme solution, the following components were combined: DMEM/F12 supplemented with 0.05 mg/mL DNase, 1 mg/mL trypsin, and 1 mg/mL collagenase IV. The enzyme solution was prepared by incubating the mixture at 37 °C for approximately 30 min. Subsequently, the tissue digestion process was conducted through enzyme washing, followed by centrifugation at 1500× *g* for 5 min. Finally, the interstitial cells were removed from the seminiferous tubules by draining the supernatant solution. After the initial enzymatic digestion process, if there were any remnants of undigested seminal tubules, they would enter the second stage of enzymatic digestion. This second stage typically lasts for approximately 15 min. All cells were extracted from the tubes at this stage. Lastly, the spermatogonial and Sertoli cells were cultured in basic culture media that contained DMEM/F12, 5% FBS, and 10 ng/mL GDNF [47]. After isolating the SSC cells from the neonate mice testicular tissue, the proportion of stem cells is 9%, which after one week of cultivation reaches 20–30% and at the end of the second week of cultivation, it reaches 70–80%, we cultured the cells for 2 weeks to increase the percentage of these cells.

#### 2.3.2. Cell Confirmation by Flow Cytometry

After two weeks of 2D culture of SSCs, confirming the identity rates of these cells was performed via flow cytometry technique using *GFRα1* and *C-Kit* markers. 4% FDA was used to fix the cells. Following fixation, 10 μL of a primary antibody, typically diluted at a ratio of 1:100, was added to approximately 10^6^ cells, and the cells with the primary antibody were then incubated at 4 °C for 1 h. After the incubation, the dish or plate containing the cells was washed twice with PBS. Next, 10 μL of a secondary antibody conjugated with FITC was added to the cells. The cells with the secondary antibody were then incubated at 4 °C for 20 min. Before flow cytometry analysis, the cells were subjected to two washes with PBS to eliminate any remaining debris or contaminants. The experiments were conducted with three replicates, ensuring the reliability and reproducibility of the results. A FC500 flow cytometer (Beckman Coulter, Miami, FL, USA) equipped with a 15-mW argon ion laser was utilized for the flow cytometry analysis. The excitation wavelength for the laser was set at 488 nm [47].

#### 2.3.3. Cell Viability Studies

To assess scaffold viability, 7 × 10^3^ SSCs were seeded per well in a 96-well plate and incubated at 37 °C with 5% CO_2_ in a humidified environment for 24 h. The extraction solution of the scaffolds was obtained according to previous studies. Briefly, the scaffolds were initially sterilized with 70% ethanol for 1 h, then rinsed with PBS solution three times. Each of the scaffolds ALGCS with different concentrations of GO (5, 15, 30, 45, and 75 µg/mL) was soaked in 1 mL cell culture medium 37 °C incubator for 1, 3, and 7 days and at predetermined time points, the culture medium in each well was replaced with 100 μL of the extraction solution specific to the ALGCS/GO scaffold. Following the replacement, an MTT solution (5 mg/mL) was added and incubated for 4 h. Finally, the solution was replaced with DMSO and incubated for 15 min. The absorbance was read at 570 nm using an ELISA reader (Bio-Rad, Hercules, CA, USA) [66].

#### 2.3.4. H&E Staining

After 7 days, scaffold samples with seeded SSCs were fixed in 10% buffered formalin and embedded in paraffin after processing. Histological cross-sections with a thickness of 5 µm were prepared from these samples. The sections were subsequently stained using the hematoxylin–eosin (H&E) staining method and examined under a light microscope [66].

#### 2.3.5. Cellular Adhesion

Cell adhesion was assessed by seeding 4 × 10^3^ cells on each well (0.5 × 0.5 cm^2^). After 48 h, the cells were stained with DAPI, and the cell number was quantified using ImageJ software V 1.8.0. (NIH, Bethesda, MD, USA).

#### 2.3.6. qRT-PCR Assay

Quantitative real-time polymerase chain reaction (qRT-PCR) was done using Rotor-Gene 6000 (Corbett Life Sciences, Sydney, Australia) to analyze gene expression. After seven days of culture, total cellular RNA from both the control and scaffold groups of SSCs was extracted using RNX-Plus solution. Subsequently, the RNA concentration and quality were assessed using a Nanodrop ND-100 UV–vis spectrophotometer (Nano-Drop Technologies, Wilmington, DE, USA) [47,69]. The cDNA was prepared using a QNRT kit. The primers are specific for glial cell-derived neurotrophic factor (*GDNF*) family co-receptor α1 (*GFRα1*). The mouse gene sequences for Inhibitor of DNA Binding 4 (*ID4*), *PLZF*, *C-Kit*, and Glyceraldehyde-3-phosphate dehydrogenase (*GAPDH*) were designed using Gene Bank as a reference and Gene Runner software (version 3.02, Hastings Software Inc., Old Tappan, NJ, USA), as shown in Table 2. *GAPDH* was used as an internal control gene. The real-time polymerase chain reaction (PCR) mixture contained 1 mL cDNA, 7 mL water, 10 mL SYBR Green qPCR MasterMix 2X, and 1 mL of 10 pmol/mL. Thermal cycling was performed for 40 cycles of 10 s at 95 °C, 10 s at 60 °C, and 20 s at 72 °C. Relative expression was reported by 2 CT method analysis.

#### 2.3.7. Immunocytochemistry Assay

The immunocytochemistry assay was performed after seven days of culture. To verify the identity of the isolated mouse SSCs, they were washed with PBS to remove residual culture media and contaminants, then fixed in a 4% PFA solution for 30 min. After the fixation process, the fixed cells were subjected to permeabilization using 0.1% Triton X-100 for 10 min. After permeabilization, the cells underwent three washes with PBS. Subsequently, the cells in ALGCS, ALGCS/GO groups were incubated with *PLZF* (diluted 1:100 in PBS) overnight at 4 °C. In the next step, the cells were incubated with fluorophore-conjugated secondary antibody diluted in 10% FBS at RT in the dark for 1 h. The secondary antibody was used as the negative control. After three times washing using PBS, the nuclei of the cells were detected using DAPI staining and then visualized using a fluorescent microscope (TE2000-S, Nikon, Tokyo, Japan) [47].

### 2.4. Statistical Analysis

Statistical analysis of the results was performed using GraphPad Prism 8 (Prism GraphPad, San Diego, CA, USA). The data were expressed as mean ± standard error (n = 3), and a one-way ANOVA test was employed for statistical comparisons. *p* < 0.05 was considered significant for all groups.

## 3. Results

### 3.1. Flow Cytometry Analysis for SSCs

**Figure 1:**
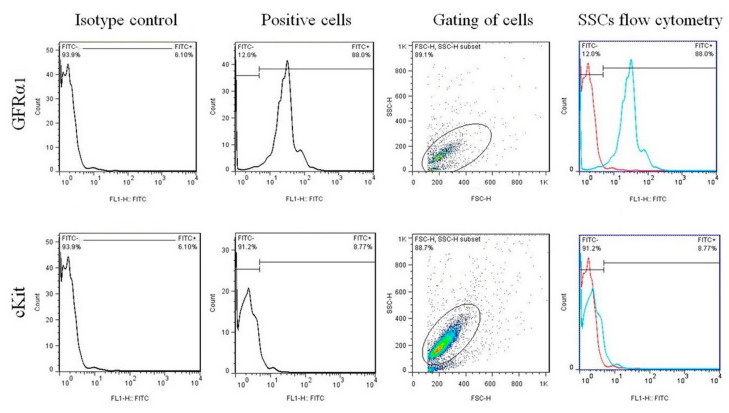
Flow cytometry analysis of surface markers *GFRα1* and *C-Kit* in isolated cells from NMRI mouse SSCs.

Flow cytometry was used to determine two surface markers for the percentage of spermatogonial stem cells, as shown in Figure 1. Flow cytometric analysis revealed an average of 88.9 ± 0.28 and 8.25 ± 0.63 of the SSCs cells express *GFRα1* and *C-Kit* on their surfaces.

### 3.2. Scaffold Characterization

#### 3.2.1. FTIR Spectra

**Figure 2:**
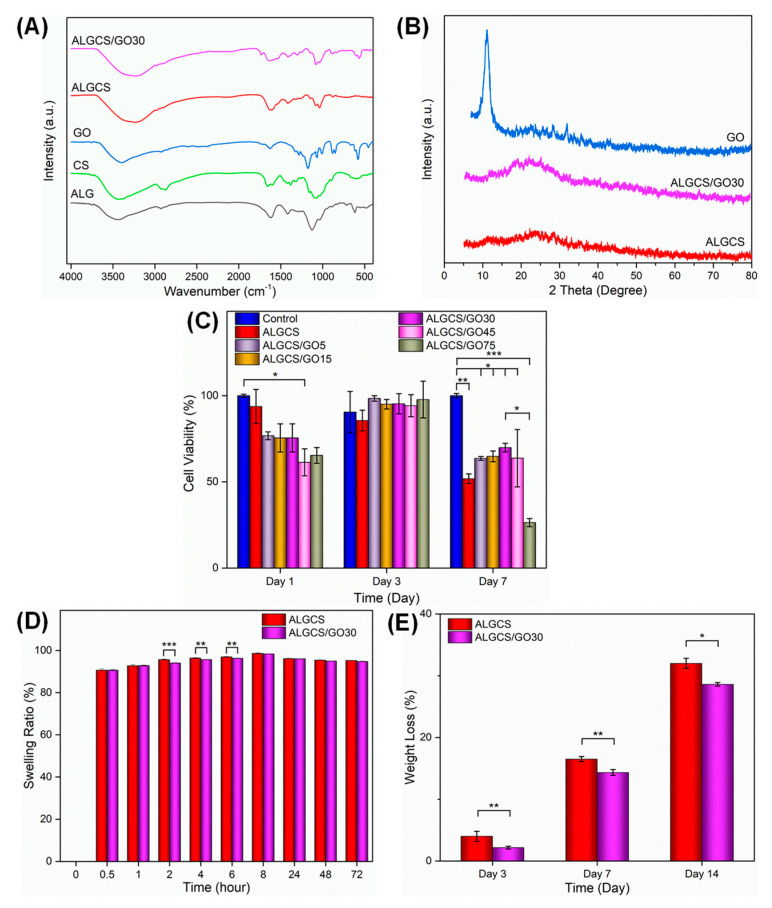
(**A**) FTIR spectra of sodium alginate (ALG), chitosan (CS), graphene oxide (GO), ALGCS and ALGCS/GO30; (**B**) XRD patterns of ALGCS, ALGCS/GO30, and GO; (**C**) MTT assay results of prepared scaffolds on SSCs. The addition of graphene oxide reduced cell viability on the scaffold, and this difference is more pronounced on day 7. However, it has been observed that the presence of 30 µg/mL graphene oxide (ALGCS/GO30) shows the highest cell viability; (**D**) the swelling percentages of the ALGCS and ALGCS/GO scaffolds in PBS at ambient temperature over time. (**E**) Weight loss percentage of different prepared hydrogels in PBS at 37 °C at different time points (3, 7, and 14 days). Values represent the mean (n = 3) ± SD. (* *p* < 0.05, ** *p* < 0.01 and *** *p* < 0.001)

Figure 2A displays the FTIR spectra of ALG, CS, GO, ALGCS, and ALGCS/GO30 nanocomposite scaffold as representatives. The FTIR spectrum of ALG revealed peaks at 3442, 1617, 1417, and 1035 cm^−1^, corresponding to OH stretching vibrations, symmetric and asymmetric stretching vibrations of COO- carboxylate salt functional groups, and C-O-C stretching vibrations, respectively [70]. The FTIR spectrum of CS exhibited peaks at 3428 cm^−1^, corresponding to OH and NH stretching vibrations and intermolecular hydrogen bonds. The peak at 1089 cm^−1^ can be attributed to C-O stretching vibrations. Additionally, the peaks at 1657, 1598, and 1321 cm^−1^ are associated with the amide I, II, and III bands, respectively [67]. The peaks at 2921 and 2874 cm^−1^ correspond to the symmetric and asymmetric stretching vibrations of the C-H group. In addition, the peaks appearing at 1423 and 1381 cm^−1^ are assigned to bending vibrations of CH_2_ and CH_3_ groups, respectively [71]. The FTIR spectrum of GO shows tensile vibrations of CO, C-OH, C=C, C=O, and OH (alcoholic and acidic) at 1069, 1285, 1629, 1725, and 3400–2400 cm^−1^, respectively [72]. In the FTIR spectrum of ALGCS, the 1623 and 1413 cm^−1^ peaks indicate that the alginate in this complex has become more deprotonated [73]. The peak observed in the 1543 cm^−1^ region can be assigned to the chitosan amide group, and the peak at 1623 cm^−1^ can be related to the overlap of the chitosan and alginate carboxyl-amide groups. The FTIR spectrum of ALGCS/GO30 shows that this sample has peaks associated with the functional groups of all three substances, chitosan, alginate, and graphene oxide, with a slight displacement and flattening, which indicates their interaction with each other due to the electrostatic interaction between the components and the interactions between the carboxyl group of GO and the –OH and –NH_2_ groups of the alginate and chitosan, as well as the formation of hydrogen bonds [67].

#### 3.2.2. XRD Analysis

The XRD pattern of GO, ALGCS, and ALGCS/GO30 nanocomposite as a representative is shown in Figure 2B. The data showed that GO has a characteristic diffraction peak (001) at 2ϴ = 10.5 with a d-spacing value of ~0.8 nm [74]. Both ALGCS and ALGCS/GO30 nanocomposite show similar trends with no clear peaks indicating their amorphous structure. Moreover, according to Bragg’s law, by increasing the distance between the GO nanosheets, the observed rise in the spectrum of nanocomposite shifted to the lower angles. The corresponding peak will be removed if the distance between the plates is too much. This trend suggests the successful dispersion of GO nanosheets in the polymer matrix, resulting in an exfoliated morphology [75]. Hence, the absence of peaks in the ALGCS/GO30 nanocomposite sample indicates that the alginate and chitosan polymers have penetrated well between the GO nanosheets and completely separated and exfoliated them. It is worth mentioning that an exfoliated structure leads to more interactions between alginate/chitosan and GO. Moreover, due to the better dispersion in exfoliated structures, GO and cells may be expected to have more exposure and higher interactions. However, the intercalated morphology of GO may lead to toughening the structure of nanocomposites [76]. We assume that in higher concentrations of GO, the intercalation and more stiffness caused by the intercalation may result in more cell death.

#### 3.2.3. Mechanical Properties

The mechanical properties of ALGCS/GO nanocomposite scaffolds are reported in Table 3. It can be seen that upon the addition of GO due to the interactions of GO with the carboxyl group of GO and the –OH and –NH_2_ groups of the alginate and chitosan, both the compression strength and modulus increase compared to the neat ALGCS. Moreover, it can be observed that in high contents, the sample becomes stiffer, which could be due to the intercalation of GO nanosheets in the structure of scaffolds, as previously confirmed by XRD results.

#### 3.2.4. Cell Viability on the Scaffolds

The cytotoxicity of the prepared samples was assessed using the MTT assay, and the results are depicted in Figure 2C. The results indicate that there were no significant differences in cell viability between the samples and the control (culture medium) on day 1 and day 3 (*p* > 0.05). This suggests that the released degradation products from the samples exhibited cytocompatibility. However, the cell viability remarkably decreased on day 7 compared to day 1 and 3. It can be seen that there is an increase in the MTT values on day 3 compared to day 1, which could be due to the initial positive effects of GO similar to CNT. However, the results revealed a significant difference in cell survival on day 7 between all samples and the control group (* *p* < 0.05). There is also a decrease in the results on day 7 compared to day 3, which could have occurred for several reasons. The first possible reason is the small area, which may have caused an overgrowth of cells, leading to cell death. The second could be that when the cells grow, they produce toxic materials, causing the death of the other cells. The third possibility could be that the cells realized the toxicity of GO more on the 7th day. The survival rate exhibited a significant increase in the ALGCS/GO30 group compared to the ALGCS group. Then, it decreased, reaching its minimum value in the ALGCS/GO75 group as the GO content increased up to 30 µg/mL. We can observe that GO at a high concentration (75 µg/mL) caused considerable toxicity as it is much closer to 100 µg/mL, which is reportedly very toxic for the cells. Therefore, according to the observed trend, the ALGCS/GO30 was considered the optimized scaffold.

#### 3.2.5. Swelling Behavior and Weight Loss

Figure 2D illustrates the swelling behavior of the cross-linked scaffolds with CaCl_2_, assessed through their water uptake values. The interactions between ALGCS chains and water significantly influence the polymer’s swelling behavior. However, the cross-linking in the ALGCS scaffold could be a protective mechanism against dissolution. The swelling behavior of ALGCS and ALGCS/GO30 scaffolds closely resemble the microenvironment of ECM, providing support for cell proliferation, differentiation, survival, and migration. The mass swelling ratios for the ALGCS and ALGCS/GO30 scaffolds were found to be 85.80 ± 30.23 and 85.39 ± 30.07, respectively. The results show that upon the addition of GO, a significant difference between ALGCS and ALGCS/GO scaffolds at 2 h (*p* < 0.0001) and 4, 6 h (*p* < 0.01) was observed, while no significant changes were observed at higher times.

Figure 2E and Table S1 illustrate the in vitro hydrolytic degradation of ALGCS and ALGCS/GO30 at 37 °C and pH = 7.4 over 3, 7, and 14 days. The findings reveal that ALGCS degrades faster than ALGCS/GO. The incorporation of GO reduces the degradation rate of the scaffold, and the differences are more pronounced on days 3 (*p* < 0.01) and 14 (*p* < 0.05).

#### 3.2.6. Morphology, Porosity, and Pore Size of the Scaffolds by SEM

**Figure 3:**
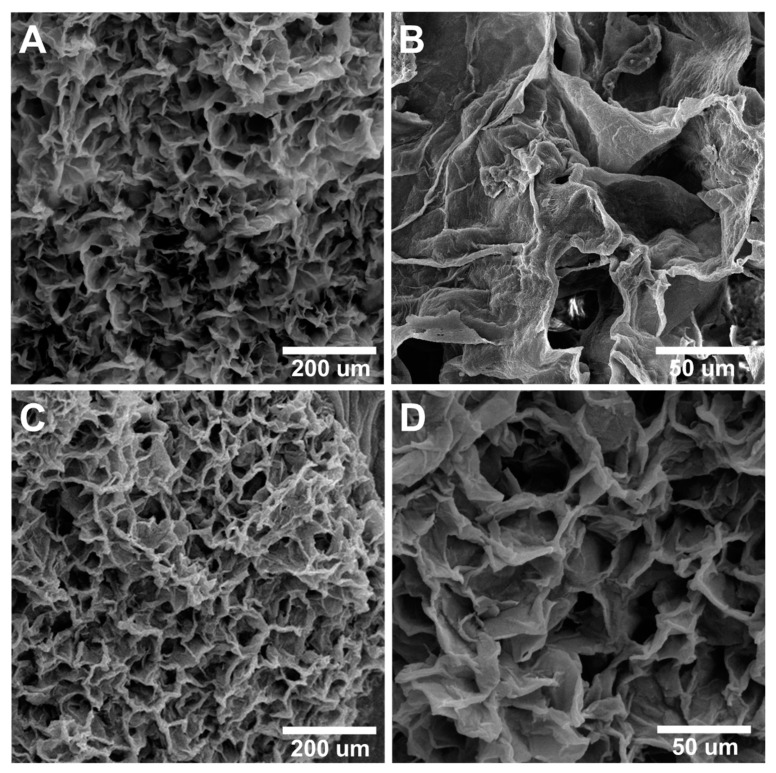
SEM images of scaffolding (**A**,**B**) ALGCS, (**C**,**D**) ALGCS/GO30. Comparison of images shows that the presence of graphene oxide increases the porosity. (Scale bar: (**A**,**C**) is 200 µm and (**B**,**D**) is 50 µm).

As depicted in Figure 3, SEM images were utilized to examine the morphology of ALGCS and ALGCS/GO30 hydrogel scaffolds as a representative, enabling the evaluation of their microstructure.

The SEM images demonstrated that all samples possess a three-dimensional structure with interconnected pores and a random orientation. The hydrogel prepared in this study displayed a highly porous structure with interconnected pores. The porosities of ALGCS and ALGCS/GO30 were calculated to be 92.58 ± 0.71% and 94.98 ± 1.25%, respectively. The results confirm that GO increases scaffold porosity; however, the differences were statistically insignificant. The ALGCS pore sizes were 40–97 μm and the ALGCS/GO30 pore sizes were in the 35–85 µm range, which is conducive for the cell attachment and proliferation of SSCs.

#### 3.2.7. Cell Adhesion and Morphology

**Figure 4:**
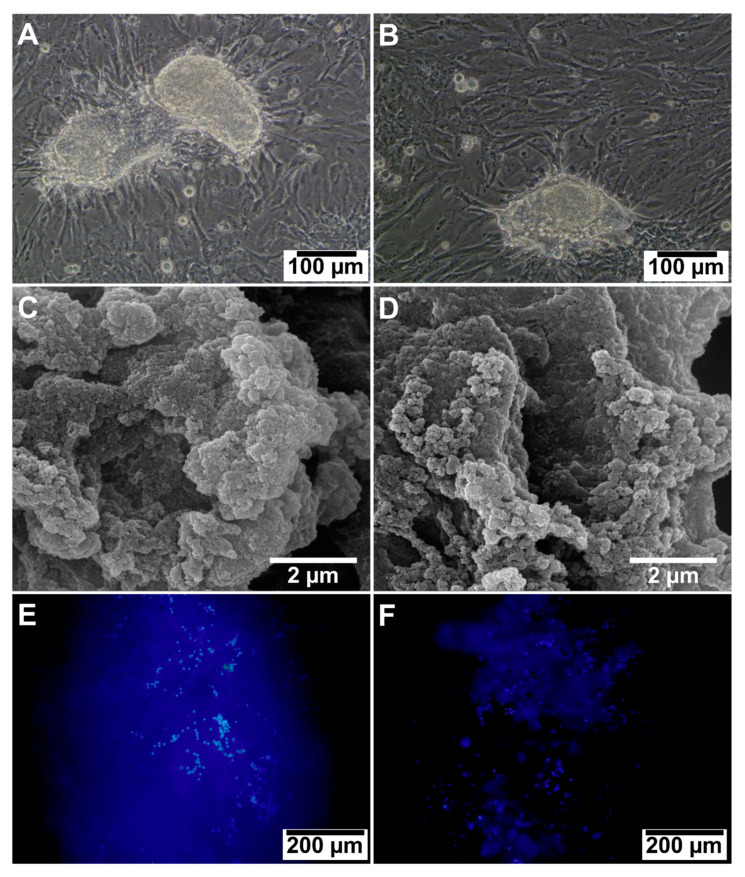
Microscopic images of spermatogonial stem cells (SSCs) on 2D in (**A**,**B**) 7 days post culture (Scale bar = 100 μm). The FESEM image of SSCs on (**C**) ALGCS/GO30 and (**D**) ALGCS. SSCs colonies are visible on scaffolds, indicating adhesion and stability (scale bar = 2 μm). DAPI staining of the nuclei (blue) of SSCs on the scaffolds (**E**) ALGCS/GO30 and (**F**) ALGCS (scale bar: 200 μm). It shows the adhesion of the cells on the scaffolds.

Figure 4A,B shows phase-contrast images of the SSCs in the 2D culture environment, depicting densely packed cells forming colony-shaped clusters. On day 7 of cell culture, SEM micrographs of the ALGCS/GO (Figure 4C) and ALGCS (Figure 4D) scaffolds displayed the adhesion and morphology of SSCs attached to their surfaces. We evaluated the adhesion and morphology of SSCs cells seeded on ALGCS and ALGCS/GO30 scaffolds using a fluorescence microscope and an inverted fluorescence microscope. To visualize the cells, staining of the cell nuclei was performed. Inverted fluorescent microscopy was used to capture DAPI staining images of cultured cells on ALGCS/GO30 and ALGCS scaffolds, as shown in Figure 4E,F. Cell counting results obtained using Image J software revealed a higher number of cells adhered to ALGCS/GO30 scaffolds compared to ALGCS scaffolds.

#### 3.2.8. H&E Staining

**Figure 5:**
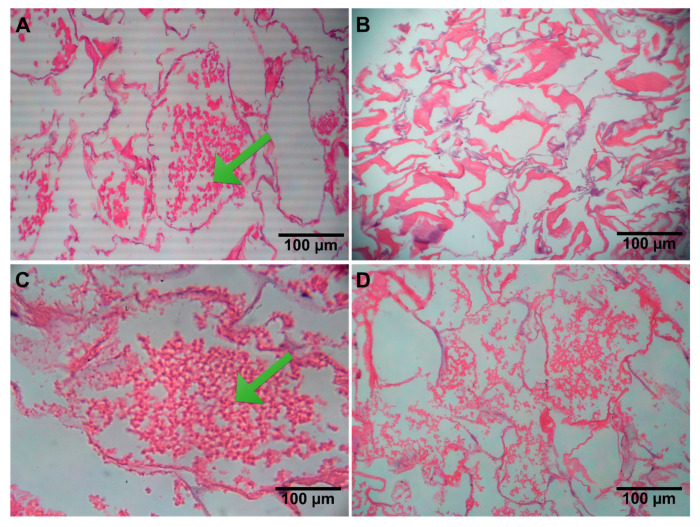
H&E-stained microscopic sections of SSCs on (**A**) ALGCS, (**B**) ALGCS free cells, (**C**) ALGCS/GO, and (**D**) ALGCS/GO free cells (scale bar: 100 μm, cell (arrowhead)).

The harvested scaffolds were stained with hematoxylin and eosin to examine cell-material interactions and confirm cell adhesion within the scaffolds. In Figure 5, the microscopic images of SSCs stained with H&E and the formation of spermatogonial colonies on ALGCS and ALGCS/GO30 scaffolds after 7 days of cell culture are presented. ALGCS/GO30 scaffold exhibited better and more abundant spermatogonial colony growth than ALGCS.

#### 3.2.9. qRT-PCR Analysis

**Figure 6:**
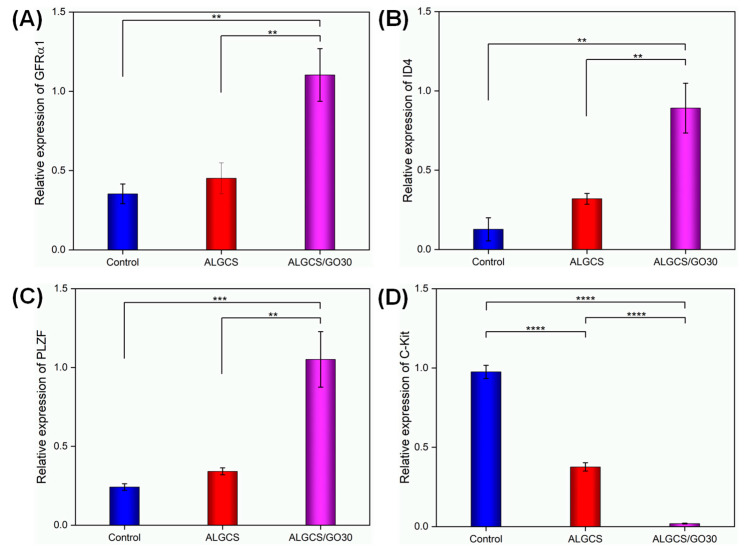
The relative expression of GFRa1, *ID4*, *PLZF*, and *C-Kit* genes of SSCs derived from colonies developed in the control and scaffolds (ALGCS and ALGCS/GO30) were analyzed by qRT-PCR. Values are expressed as means ± SD, three samples were examined for each group, and all evaluations were performed in three replications. (**A**) Expression of GFRa1, (**B**) expression of *ID4*, (**C**) expression of *PLZF*, and (**D**) expression of *C-Kit* (** *p* < 0.01, *** *p* < 0.001 and **** *p* = 0.00001).

Figure 6 illustrates the comparison of proliferation levels of SSCs between the 2D control group and the 3D ALGCS and ALGCS/GO30 groups. The qRT-PCR analysis indicated that ALGCS/GO30 exhibited significantly higher expression levels of SSC genes, including *GFRα1* and *ID4*, compared to both ALGCS and the control group.

Figure 6A depicts the expression levels of the *GFRα1* gene in the SSC cell line treated with ALGCS and ALGCS/GO30. Comparatively, the expression levels of *GFRα1* in the SSCs exposed to both study groups were significantly higher than in the control group (*p* < 0.01). However, ALGCS/GO30 displayed more *GFRα1* gene expression on cells than ALGCS (*p* < 0.01). Figure 6B displays the expression levels of the *ID4* gene in SSCs treated with ALGCS and ALGCS/GO30. The *ID4* gene expression levels in the two study groups were significantly higher than the control group (*p* < 0.01), and ALGCS/GO30 exhibited significantly higher *ID4* gene expression compared to ALGCS (*p* < 0.01). As depicted in Figure 6C, the *PLZF* gene expression levels in SSCs treated with both ALGCS and ALGCS/GO30 were significantly higher than in the control group (*p* < 0.001). Furthermore, ALGCS/GO30 demonstrated significantly higher *PLZF* gene expression in SSCs than ALGCS (*p* < 0.01). Figure 6D shows that the gene expression of *C-Kit* in the scaffolds decreased compared to the control group. Notably, ALGCS/GO30 exhibited a significant reduction in *C-Kit* gene expression (*p* = 0.00001) compared to both ALGCS and the Control groups.

#### 3.2.10. Immunocytochemistry

**Figure 7:**
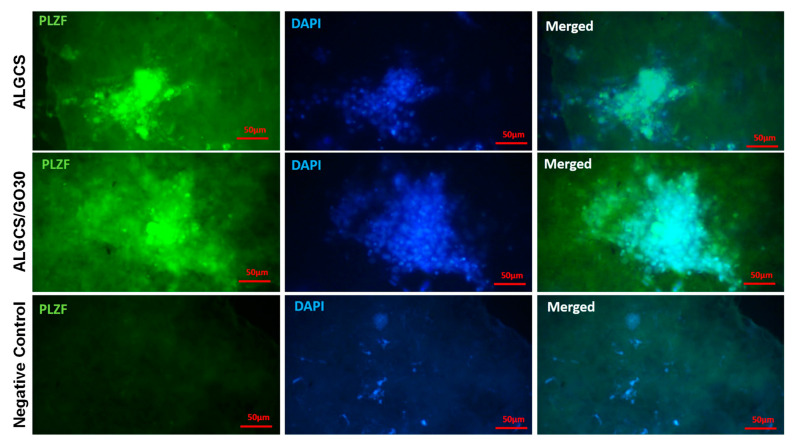
Immunofluorescent spermatogonial stem cells (SSCs) staining in 3D cell culture after 7 days post treatment. The *PLZF* marker with FITC is in the first column, DAPI staining is in the second column, and a merging of FITC and DAPI is in the third column in each experimental group. Scale bar = 50 µm.

To analyze the expression of proliferation markers of SSCs, an immunocytochemistry test was performed within 7 days. In all samples (ALGCS, ALGCS/GO30, and Negative Control), the expression of *PLZF* (FITC- and DAPI-stained) as an undifferentiated marker was observed, confirming its presence in both ALGCS and ALGCS/GO30 samples (Figure 7). Although the number of cells in negative control was equal to the other groups, no color reaction was seen because the primary antibody was not added to this group. In Figure S2, the expression of *PLZF* in ALGCS/GO30 was 73.68 ± 2.38, significantly higher than ALGCS, which had an expression of 55.10 ± 2.00 (*p* < 0.001).

## 4. Discussion

Spermatogenesis is a crucial biological process in which animals generate spermatozoa from spermatogonial stem cells (SSCs). The successful in vitro differentiation of SSCs into spermatids offers promising prospects for regenerating impaired spermatogenesis. The study findings demonstrated that SSCs exhibited survival and proliferation capabilities on both ALGCS and ALGCS/GO scaffolds. However, the ALGCS/GO group showed the improved colonization, proliferation, and gene expression of SSCs compared to the ALGCS and control groups. In our study, a scaffold was designed to mimic the non-topographic features of the base membrane to enhance the efficiency of in vitro spermatogenesis. In addition to possessing favorable biological features, a scaffold should also exhibit biomechanical characteristics that are similar to the cells it supports to promote their proliferation and differentiation effectively [77]. Among the various natural and synthetic materials available, alginate and chitosan have emerged as widely used bio-scaffolds due to their remarkable properties, including excellent biological characteristics, outstanding mechanical structure, and minimal inflammatory reactions [78]. GO is acknowledged as a class of nanomaterials with immense potential in numerous biomedical applications, primarily due to its distinctive features, such as excellent mechanical stability and its ability to enhance scaffolds’ overall strength and durability when combined with other materials [79]. GO has demonstrated promising results in promoting the proliferation and differentiation of various stem cell types, indicating its potential in advancing regenerative medicine and tissue engineering applications [80]. The GO concentrations of (5, 15, 30, 45, and 75 µg/mL) were added to ALGCS, and the MTT test was performed on 1, 3, and 7 days, revealing an adequate amount of difference between ALGCS/GO30 and ALGCS/GO75 on the 7th day, also manifesting the optimized dose for these cells as 30 µg/mL. GO was reported to induce cell toxicity through plasma membrane damage, the generation of ROS, and DNA damage [81]. However, our study yielded interesting results indicating that GO concentrations up to 30 µg/mL can be beneficial for cell attachment and proliferation. The scaffolds’ properties were thoroughly assessed through degradation and swelling tests. The findings unveiled that the ALGCS scaffold exhibited higher swelling than the ALGCS/GO30 scaffold, underscoring the impact of GO on mitigating the degree of swelling in the composite scaffold. This difference in swelling behavior can be attributed to the presence of GO in the ALGCS/GO30 scaffold, which contributes to a reduction in swelling. The presence of GO introduces hydrophobic characteristics to the scaffold. Hydrophobic GO has the ability to interact with and absorb various organic molecules or polymers through van der Waals interactions [82]. Also, given that sufficient differences in the ALGCS/GO30 alliance were observed in 2, 4, and 6 h, respectively. It can be stated that the degradation rate of ALGCS scaffolding was higher compared to ALGCS/GO30 because GO causes mechanical stability and strength for the scaffold. In addition, the slow degradation of ALGCS/GO30 scaffolds are suitable for SSC tissue engineering. A porosity analysis was also carried out on scaffolds, which revealed that the porosity of the ALGCS/GO30 scaffold was higher than ALGCS because GO leads to an increase in the ratio of area to volume [83].

This study utilized SSCs isolated from 3- to 6-day-old NMRI mice. Through flow cytometry, we were able to identify that these cells were in the proliferating phase, laying the foundation for our subsequent investigations. Moreover, we evaluated the effects of GDNF on SSCs. Although specific and exclusive markers for SSCs are not well-defined, evaluating the expression of multiple markers can provide valuable information about these cells in individuals [84]. To confirm the presence of undifferentiated or differentiated spermatogonial cells during cultivation, we performed qRT-PCR tests using specific markers associated with spermatogonia. In order to confirm the presence of undifferentiated spermatogonial cells during cultivation, we conducted a qRT-PCR test using specific markers associated with spermatogonia, including *ID4* and *GFRα1*. In addition to evaluating the expression levels of the differentiating SSCs marker *C-Kit*, undifferentiated spermatogonial cells were confirmed by assessing the expression levels of specific markers *ID4* and *GFRα1* in all culture groups. These markers are widely recognized as indicators of spermatogonial stem/progenitor cells in various species. These markers play a crucial role in identifying and characterizing undifferentiated spermatogonial cells. The expression of the *C-Kit* marker is known to be low or absent in A_s_, A_pr_, and early A_al_ spermatogonial cells, indicating their undifferentiated state. However, *C-Kit* expression increases significantly in late A_al_ and further differentiated spermatogonial cells, signifying their differentiation [85]. In our study, the expression of *GFRα1*, *ID4*, and *PLZF* markers of spermatogonial were perceived in all groups. Our findings also signified that *GFRα1*, *ID4*, and *PLZF* gene expressions were expressed more in ALGCS/GO30 than in the control and ALGCS groups, which agrees with previous studies [47,49,60,62,86]. The *C-Kit* gene is expressed in the early stages of meiosis, and a decrease in its expression can indicate increased proliferation of SSCs [87]. As can be seen in the C-Kit expression results, the expression of gene in the scaffolds were much lower than the control group, and ALGCS/GO30 has the lowest expression, which indicates the proliferation of SSCs in scaffolds were higher than in the control group. At the end of the proliferative period in the culture, the scaffold group exhibited a higher level of expression for the *ID4*, *PLZF*, and *GFRα1*, and a lower level of expression for the *C-Kit* genes compared to the control group. The differences in gene expression were found to be statistically significant in all the results (*p* < 0.05). The immunocytochemical study conducted in our research corroborated the results obtained from the qRT-PCR results. The analysis also revealed the increased immunoreactivity of the *PLZF* marker in the ALGCS/GO30 scaffold. The results demonstrated that freeze-dried ALGCS/GO30 scaffold supports the proliferation and colonization of SSCs. The nanocomposite scaffolds possess excellent mechanical properties and strength. With their impressive mechanical properties and microstructure, these scaffolds closely mimic the natural ECM microenvironment. We believe that as in the prepubertal boys, the testis lacks mature sperm, freezing the biopsied testicular tissue, cell suspension in the hope of future auto transplantation or the in vitro maturation of the germ cells could be an alternative. Consequently, the nanocomposite scaffolds hold significant potential to support the proliferation and differentiation of SSCs, making them promising candidates for applications in both experimental and clinical settings.

## Figures and Tables

**Table 1:** Scaffold preparations and groups.

Abbreviations	Scaffolds
ALGCS	Alginate/chitosan
ALGCS/GO5	Alginate/chitosan + GO concentration 5 µg/mL
ALGCS/GO15	Alginate/chitosan + GO concentration 15 µg/mL
ALGCS/GO30	Alginate/chitosan + GO concentration 30 µg/mL
ALGCS/GO45	Alginate/chitosan + GO concentration 45 µg/mL
ALGCS/GO75	Alginate/chitosan + GO concentration 75 µg/mL

**Table 2:** The sequence of forward and reverse primers of the qRT-PCR genes.

Gene Name	Symbol	Accession Number	Forward/Reverse
GDNF family receptor alpha 1	*GFRα1*	NM_001285457.2	TCAGATATATTCCGGGCAGTCC CATCGAGGCAGTTGTTCCCT
Inhibitor of DNA binding 4	*ID4*	NM_031166.3	TGAAGCAGCAGTTGACATCTCT GGACACAGGCAATATCCTCATAGAA
Proto-oncogene C-Kit	*C-Kit*	XM_021163091.1	CTAAAGATGAACCCTCAGCCT GCATAACACATGAACACTCCA
glyceraldehyde-3-phosphate dehydrogenase	*GAPDH*		CCCTTAAGAGGGATGCTGCC GTTCACACCGACCTTCACCA
Promyelocytic leukemia zinc finger	*PLZF*	NM_001289726.1	CCCGTTGGGGGTCAGCTAGA CTGCAAGGTGGGGCGGTGTAG

**Table 3:** The mechanical properties of the hydrogels.

Samples	Compression Strength	Modulus
	(MPa)	(MPa)
ALGCS	0.153 ± 0.11	0.754 ± 0.07
ALGCS/GO30	0.185 ± 0.09	0.856 ± 0.09
ALGCS/GO75	0.267 ± 0.13	0.911 ± 0.11

## Data Availability

The data presented in this study are available in the article and supplementary material.

## Supplementary Materials

The following supporting information can be downloaded at: https://www.mdpi.com/article/10.3390/jfb14120556/s1, Figure S1: (A) Appearance of alginate and chitosan scaffolds synthesized with different percentages of graphene oxide (5, 15, 30, 45, 75 µg/mL), (B) Graphene oxide solution 1 mg/mL, which was used in the preparation of scaffolds. Figure S2: The expression of PLZF on ALGCS, ALGCS/GO30, a significant difference between ALGCS/GO30 and ALGCS (*** *p* < 0.001). Table S1: Degradation of the scaffolds.
