# Supplementary material for: The Effect of Chitosan/Alginate/Graphene Oxide Nanocomposites on Proliferation of Mouse Spermatogonial Stem Cells
Source: J Funct Biomater. 2023 Nov 22;14(12):556. doi: 10.3390/jfb14120556 (PMC10744091; doi:10.3390/jfb14120556)
Supplement: Supplementary file 1 [file jfb-14-00556-s001.zip › jfb-2683262-supplementary.pdf]

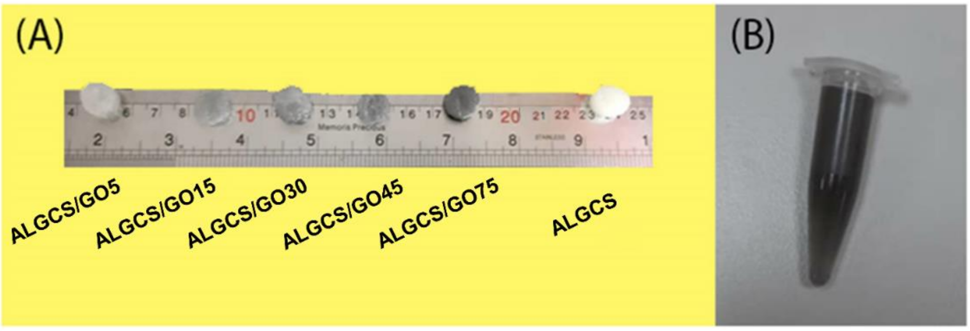

Figure S1. (A) Appearance of alginate and chitosan scaffolds synthesized with different percentages of graphene oxide (5, 15, 30, 45, 75 μg/mL), (B) Graphene oxide solution 1 mg/mL, which was used in the preparation of scaffolds.

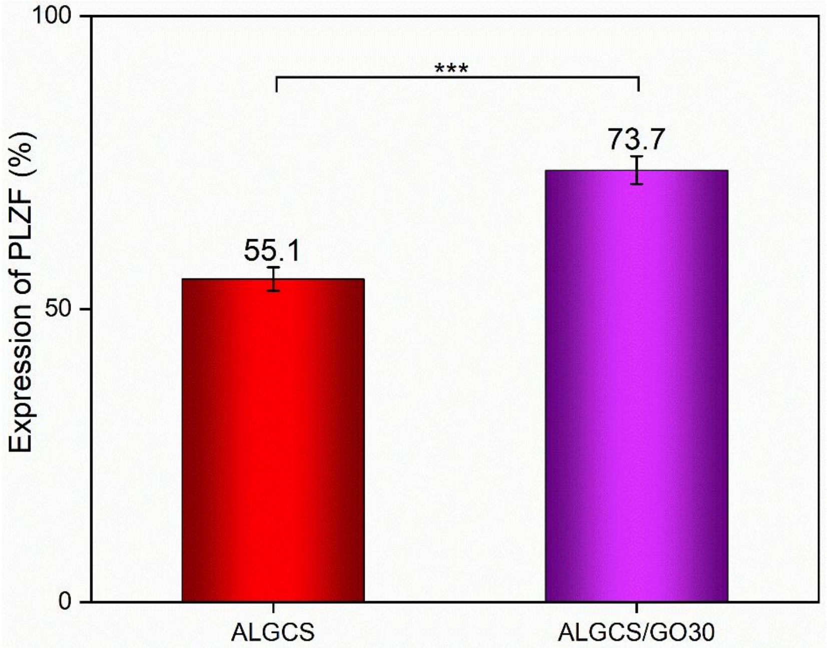

Figure S2. The expression of PLZF on ALGCS, ALGCS/GO30, a significant difference between ALGCS/GO30 and ALGCS (*** $p < 0.001$).

Table S1. Degradation of the scaffolds.

Samples	Degradation day 3	Degradation day 7	Degradation day 14
ALGCS	4.01 ± 0.82	16.50 ± 0.41	32.12 ± 0.82
ALGCS/GO30	2.17 ± 0.24	14.33 ± 0.47	28.60 ± 0.29
